# Narrative Length and Speech Rate in Battered Women

**DOI:** 10.1371/journal.pone.0142651

**Published:** 2015-11-10

**Authors:** Violeta Fernández-Lansac, María Crespo

**Affiliations:** 1 Universidad Complutense de Madrid, Facultad de Psicología, Campus de Somosaguas, Madrid, Spain; 2 Universidad Complutense de Madrid, Facultad de Psicología, Campus de Somosaguas, Madrid, Spain; University of Florida, UNITED STATES

## Abstract

Narrative length and speech rate of traumatic recollections have been previously associated with different emotions and adjustment trajectories after trauma. However, the evidence is limited and the results are mixed. The present study aimed to evaluate length (i.e., word count) and speech rate (i.e., words per minute) in narratives of events with different valence (i.e., neutral, positive, and negative/traumatic) by 50 battered women (trauma group) and 50 non-traumatized women (controls). The results showed that traumatic narratives by the trauma group were longer than those by the control group. Moreover, they were inversely related to time since the event and anxiety during disclosure, whereas the speech rate was also inversely associated with anxiety, as well as with peritraumatic dissociation and avoidance. The shorter narratives for positive events and a decelerated speech pattern for traumatic experiences predicted psychological symptoms. Additionally, the individual’s emotional state predicted narrative aspects, with bidirectional effects. Our findings showed that linguistic characteristics of traumatic narratives (but also of narratives of positive events) revealed information about how the victims elaborated autobiographical memories and coped with the trauma.

## Introduction

The nature of trauma narratives and their relationship with posttraumatic symptoms have received growing interest aimed to prove psychological mechanisms involved in the memories of traumatic events and in the development of posttraumatic stress disorder (PTSD). Narrative studies focus on establishing the connection between adjusting after trauma and integrating the memory of the event within the autobiographical memory system (for a review, see [1,2]).

Among the structural aspects of traumatic memories, *narrative length* has received attention because it is considered to reveal particular aspects of psychological functioning after a traumatic experience [3,4]. It is widely known that emotional events are better remembered than non-emotional ones, and it has been observed that an elevated emotional response can lead to an enhanced memory [5,6]. As a result, high arousal might result in longer trauma narratives [4, 7, 8]. Furthermore, due to the salience of trauma in personal life history or event centrality, it has been generally assumed that trauma narratives tend to be longer and more detailed compared to other life narratives [9–11]. Moreover, several authors have observed a global decrease in length and factual details of trauma narratives over time, likely due to normal processes of decay that make memories less accessible [12, 13]. This observation suggests that time since the event might also influence the narrative length.

Alternatively, information-processing theories [14–16] indicate that high arousal during trauma could affect the encoding process through mechanisms such as peritraumatic dissociation, causing difficulties in elaborating a declarative representation of the event. Therefore, the victims will initially have difficulties in expressing the traumatic event. Over time, they will be able to elaborate a coherent narrative regarding the traumatic experience [17]. According to this hypothesis, trauma narratives (initially at minimum) are predicted to be shorter due to both difficulties in encoding and recalling processes of highly emotional and salient memories.

Related to these two perspectives, there are also discrepancies among the authors regarding the relationship between trauma narrative length and psychological adjustment [3–4]. Dekel and Bonanno [13] found a moderate association between length of narratives in two different time points and PTSD, and Alvarez-Conrad et al. [18] noted a positive correlation between the word count for pre-threat sections of trauma narratives (before the first expression of perceived danger) and physical symptoms and discomfort. Additionally, the results obtained by Römisch et al. [8] demonstrated a relationship between narrative length and intrusion (they considered length as an indicator of immersion, which is related to the intense and often involuntary reliving of trauma in PTSD).

Conversely, Beaudreau [4] found that longer trauma narratives were associated with a better adjustment, whereas shorter narratives could indicate repression and avoidance mechanisms. In fact, Lindblom and Gray [11] showed that avoidance symptoms of PTSD predicted trauma narrative length, and Gray and Lombardo [9] explained that anxiety during disclosure could result in avoidance of event details. Consistent with those findings, Foa et al. [19] analyzed the changes in trauma narrative by sexual assault victims over the course of exposure therapy, and they found an increase in narrative length during treatment that likely revealed an improvement in trauma elaboration together with decreased anxiety. However, the authors noted that the findings could also be explained because therapy used the repeated imaginal reliving, which could produce hypermnesia or increased recall. Beaudreau [4] concluded that whereas memory elaboration was often considered to be crucial for the recovery of victims, over-elaboration of the event could also be associated with a worse adjustment.

Furthermore, it is worth mentioning the limited attention paid in previous studies to the *speech rate* of trauma narratives, although it could also reveal important information regarding memory processing and emotions. Specifically, a decrease in the speech rate has been associated with sadness and depression, and an accelerated speech has been associated with anxiety, and changes in the voice-style have proven to affect cardiovascular response [20]. Additionally, pauses have been considered to be an indicator of narrative fragmentation because they might reflect difficulties in elaborating and planning, as well as indicate a high arousal during recall [8]. Also, it has been suggested that long pauses could be related to a dissociative response [21]. These factors suggest that the differences in speech rate might be associated with different emotional responses to the traumatic experience.

In sum, evidence regarding the narrative length of trauma memories is fragmented and controversial, and speech rate has hardly been considered. In fact, aspects such as narrative skills, strategies for addressing stress, or willingness to disclose likely have a considerable effect on how individuals report a negative experience [22]. Therefore, the objective and subjective features of the event, and of the response to the event, must be explored in future studies. Summarizing previous findings, the time since the traumatic experience has proven to be an important objective aspect, whereas event centrality and peritraumatic dissociation likely impact memory processing and subsequently the traumatic narratives. To address trauma memory, strategies such as avoidance or memory elaboration have been highlighted in relationship with narrative length. It is also expected that narrative construction will be affected by the frequency of talking about the event and the use of psychological assistance. Additionally, the narrative expression will likely be related to the degree of anxiety suffered during disclosure. Furthermore, the relationship between narrative length and speech rate with PTSD should be clarified, but also with other psychological symptoms.

### Objectives

The present study aimed to explore the following: (1) narrative length and speech rate in traumatized (trauma group) and non-traumatized subjects (controls) (after controlling narrative skills) across positive, and negative or traumatic events (i.e., different valence); (2) relationships between the trauma narrative length and the speech rate, and several objective and subjective features of the traumatic event and coping mechanisms (e.g., time since the occurrence of the event, anxiety during disclosure, centrality for identity, peritraumatic dissociation, elaboration, and avoidance); and (3) relationship between the trauma narrative length and the speech rate, and psychological adjustment (i.e., PTSD, depression, and anxiety symptoms).

## Methods

The Ethics Committee of the University approved this study and the informed consent. All of the participants provided their written informed consent.

### Participants

Fifty traumatized women (trauma group) and 50 non-traumatized women (control group) participated in the study. All of the participants were aged ≥18 years and were fluent in Spanish. Participants from the trauma and the control group were matched by age, according to three age ranges (18–34, 35–54, and 55–74 years). The participants from the trauma group were recruited by clinics and centers that assist battered women. All of them had suffered violence by their intimate partners for at least 1 month. Referring counsellors evaluated the participants and considered them to be emotionally able to withstand the session. The control group was recruited by word-of-mouth advertising. All of the participants received a compensation of 15 €.

Power analyses using G-Power software showed that *n* = 90 was needed to achieve a desirable power over .80 (medium-size effect = .30; alpha = .05).

### Procedure

The assessment was conducted in a 1.5- to 2-hr session. After completing a semi-structured interview using different self-administered instruments, the participants were asked to narrate a normal day in their life (*neutral*), the most distressing episode of maltreatment (trauma group) or the most stressful event (controls) (*traumatic/negative*), and the happiest event (*positive*). The order of episodes (negative and positive) was randomly counterbalanced across participants, although the neutral narrative was invariantly the first. They were requested to accurately recall the events, providing as much detail as possible. When the negative event, primarily the traumatic event, was the last, emotional support strategies were used to minimize any discomfort after the session. After each narrative, the participants were asked to rate their anxiety during disclosure using a 0–100 point visual-analogue scale. The narratives were uninterrupted, audio-recorded, and then transcribed verbatim. The assessment protocol was previously tested and improved using a pilot study with five psychology students.

### Measures

#### Demographic variables and verbal intelligence

A standardized interview assessed background information (e.g., age, education, and socioeconomic level), and details of the traumatic/negative event (e.g., time of the event and injuries), and psychological treatment (e.g., months in therapy). In this interview, the participants were also asked to select the worst stressful event in their life (control group) or the worst episode of violence inflicted by their intimate partner (trauma group).

Verbal intelligence was measured using the Vocabulary subtest of the Wechsler Adult Intelligence Scale-III (WAIS-III-V) [23] in which the participants provided oral definitions for 33 words presented. In this sample, Cronbach’s alpha was .91.

#### Psychological symptoms

The Global Assessment of Posttraumatic Stress Questionnaire (Evaluación Global del Estrés Postraumático (EGEP) in Spanish) [24] is a self-reported measure that allows the evaluation of posttraumatic symptoms in several domains. It provided information on the presence and severity of symptoms according to the DSM-IV-TR [25] criteria and PTSD diagnostic. In this study, it showed an adequate consistency for the severity of symptoms (alpha = .94).

The Beck Depression Inventory-II (BDI-II) [26] is likely the most widely used test of depressive symptoms. In this study, Cronbach’s alpha was .94.

Similarly, the Beck Anxiety Inventory (BAI) [27] is commonly used to measure the presence and the severity of anxiety symptoms. In this sample, it showed high internal consistency (alpha = .95).

#### Characteristics of negative/traumatic event and its memory

The Centrality of Event Scale (CES) [28] measures the extent to which a traumatic memory forms a central component of personal identity and a reference point for the attribution of meaning to other experiences in a person’s life. Its reduced 7-items version was used. It demonstrated good internal consistency in this study (alpha = .91).

The Autobiographical Memory Questionnaire (AMQ) [29] is a series of questions concerning the processes involved in remembering an event. For this study, two questions regarding avoiding thinking of the event and memory elaboration were selected for their relevance to our goals.

The Peritraumatic Dissociative Experiences Questionnaire-Self Report version (PDEQ) [30, 31] assesses the degree of dissociative experience at the time of the stressful event. In this study, internal consistency was satisfactory (alpha = .90).

#### Narrative aspects measures

Calculating the narrative length was achieved using the Linguistic Inquiry and Word Count (LIWC) software [32]; Spanish adaptation by Ramírez-Esparza et al. [33]. LIWC is a text analysis software program for measuring the presence of emotion words, cognitive processes, and other linguistic characteristics of the spoken or written language. In the present study, only word count (WC) was considered. Nonetheless, the speech rate was determined by dividing the number of words (word count) by the narrative duration (minutes), obtaining the number of words per minute (W/min.) for each narrative.

### Data Analyses

The descriptive statistics (i.e., means, standard deviation, and percentages) were used to characterize the sample and study variables. The chi-squared tests for the categorical data and *t* test or Mann-Whitney *U* tests for the continuous data were applied to compare the trauma and control groups, and anxiety during the disclosure of narratives with difference valence. The data were tested for normality using the Shapiro-Wilk test. The relationships between the narrative length and speech rate were explored using Pearson and point-biserial correlations. Additionally, both variables were correlated with the objective and subjective aspects of traumatic memories, coping strategies, and anxiety during disclosure.

The differences between the narrative length and speech rate were analyzed using analyses of covariance (ANCOVAs) for repeated measures, with *group* (trauma vs. control group) as the between-subject factor and *narrative valence* (positive vs. traumatic/negative) as the within subject factor (2 × 2 design). The narrative length and speech rate for the neutral event were introduced as the covariance terms to control for potential confounds. Specifically, it allowed the narrative style to be controlled. Verbal intelligence was not considered as a covariance after being explored. The Bonferroni correction was used to adjust the confidence intervals.

Finally, multiple linear regression analysis was used to examine whether the narrative length and speech rate for the positive and traumatic narratives explained the presence of psychological symptoms in the trauma group. Previously, Pearson correlations were examined. Because a bidirectional influence was expected, regression was also employed to examine whether the psychological aspects predicted the four narrative variables. In the distribution exploratory analysis, the quadratic relationships between variables were discarded. The stepwise discriminant function analysis was used to select the best predictors from the linear regression.

## Results

### Participants and negative/traumatic event characterization

The participants from the trauma group were, for the most part, Spanish (82.0%), and 18.0% of the participants were Latin American; ages ranged between 21 and 60 years (*M* = 40.46; *SD* = 9.27). The control group was 92.0% Spanish (8.0% were Latin American), with ages ranging between 20 and 73 years (*M* = 38.82; *SD* = 14.48). Both groups did not differ in nationality, educational level, or job situation; however, the groups did differ slightly in marital status (20.0% of the trauma group and 38.0% of the controls were married or living with their partners). This difference was justified given the specific nature of the traumatic experience in the trauma group. When verbal intelligence was analyzed, the results showed that there were no significant differences between the trauma and control groups in WAIS-III-V. Table 1 shows the most important demographic variables for participants from the two groups.

**Table 1:** Sociodemographic features of participants by group.

Variables	Trauma Group (*n* = 50)	Control Group (*n* = 50)	Statistics	*p*
Nationality: Spanish *% (n)*	82.0 (41)	92.0 (46)	*χ* ^*2*^(1, 100) = 2.21	.137
Age (years) *M (SD)*	40.46 (9.27)	38.82 (14.48)	*Z* = -.821	.412
Educational level: High school degree (vs. compulsory education) *% (n)*	64.0 (32)	74.0 (37)	*χ* ^*2*^(1, 100) = 1.17	.280
Job situation: Active (vs. inactive)	54.0 (27)	58.0 (29)	*χ* ^*2*^(1, 100) = .162	.687
Marital status: No partner (vs. with partner) *% (n)*	20.0 (10)	38.0 (19)	*χ* ^*2*^(1, 100) = 3.93	.047
Months since negative/traumatic event *M (SD)*	69.18 (79.13)	107.71 (110.66)	*Z =* -1.727	.084
Verbal intelligence (WAIS-V) *M (SD)*	40.17 (11.34)	40.08 (10.39)	*Z* = -.197	.844

The participants from the trauma group had suffered violence by their intimate partner during a mean of 136.70 months (*SD* = 133.06), that is, over 11 years. Approximately 62.0% suffered physical aggressions in the worst episode of violence that they identified, 12.0% suffered sexual abuses, and all of them suffered psychological abuses (100.0%). Only 10.0% from the control group selected an aggression as the worst event lived. The most common events reported were the death or illness of a loved one (58.0% and 16.0%, respectively). Other events included family abuse, job problems, abortion, or eviction. The difference between groups in the time distance of the negative/traumatic event was not statistically significant (see Table 1).

### Anxiety during disclosure

In general, there was a high variability among anxiety perceived during disclosure by the participants. Between the groups, there were significant differences in anxiety reported for the neutral narrative (*Z* = -3.469, *p* = .001), for the positive narrative (*Z* = -2.540, *p* = .011), and for the negative narrative (*Z* = -4.761, *p* < .001). For the three narratives, the trauma group showed more anxiety, especially when the participants narrated the traumatic event. The means for the trauma group were 29.29 (*SD* = 30.05), 21.94 (*SD* = 29.26), and 72.45 (*SD* = 92.03), respectively; for the control group, the means were 10.10 (*SD* = 15.73), 7.72 (*SD* = 16.13), and 41.90 (*SD* = 29.33), respectively.

### Narrative length and speech rate comparisons

In the first step, the correlations between the narrative length and speech rate for the neutral, positive, and negative/traumatic events were explored (Table 2). All of the variables correlated directly and significantly with each other, with the exception of the neutral narrative length and the speech rate for the positive and negative/traumatic narratives. The results suggest that, although the speech rate and narrative length were associated, they measured different narrative aspects.

**Table 2:** Correlations between narrative length (WC) and speech rate (W/min.) for neutral, positive and negative/traumatic narratives (*n* = 100).

	WC Neutral	WC Positive	WC Negative/Traumatic	W/min. Neutral	W/min. Positive
**WC Positive**	.526				
	*p* < .001				
**WC Negative/Traumatic**	.273	.279			
	*p* = .006	*p* = .005			
**W/min. Neutral**	.412	.359	.220		
	*p* < .001	*p* < .001	*p* = .028		
**W/min. Positive**	.159	.443	.342	.570	
	*p* = .114	*p* < .001	*p* = .001	*p* < .001	
**W/min. Negative/Traumatic**	.135	.261	.453	.476	.660
	*p* = .181	*p* = .009	*p* < .001	*p* < .001	*p* < .001

In line with previous studies (e.g., Beaudreau [4]), there was a wide variability in the narrative length and speech rate among participants in the three different narratives. The mean word count for the neutral narratives was 266.38 (*SD* = 227.93), and that for words per minute was 144.71 (*SD* = 27.85) for the trauma group; for the controls, the mean word count was 200.34 (*SD* = 234.19) and that for words per minute was 130.56 (*SD* = 25.14). The descriptive characteristics for the positive and negative/traumatic narratives are shown in Table 3.

**Table 3:** Comparison between groups and narrative valence for narrative length (WC) and speech rate (W/min.): mean (standard deviation) (*n* = 100).

	Trauma Group (*n* = 50)		Control Group (*n* = 50)		*F*(1,97)		
Variables	Positive	Traumatic	Positive	Negative	Group	Valence	Group × Valence
WC	235.10	854.78	177.88	391.48	12.644	23.704	12.177
	(267.12)	(631.13)	(171.39)	(518.36)	*p* = .001	*p* < .001	*p* = .001
W/min.	141.36	145.81	127.82	130.17	2.092	2.053	.511
	(32.41)	(32.37)	(26.58)	(26.94)	*p* = .151	*p* = .155	*p* = .476

**Fig 1:**
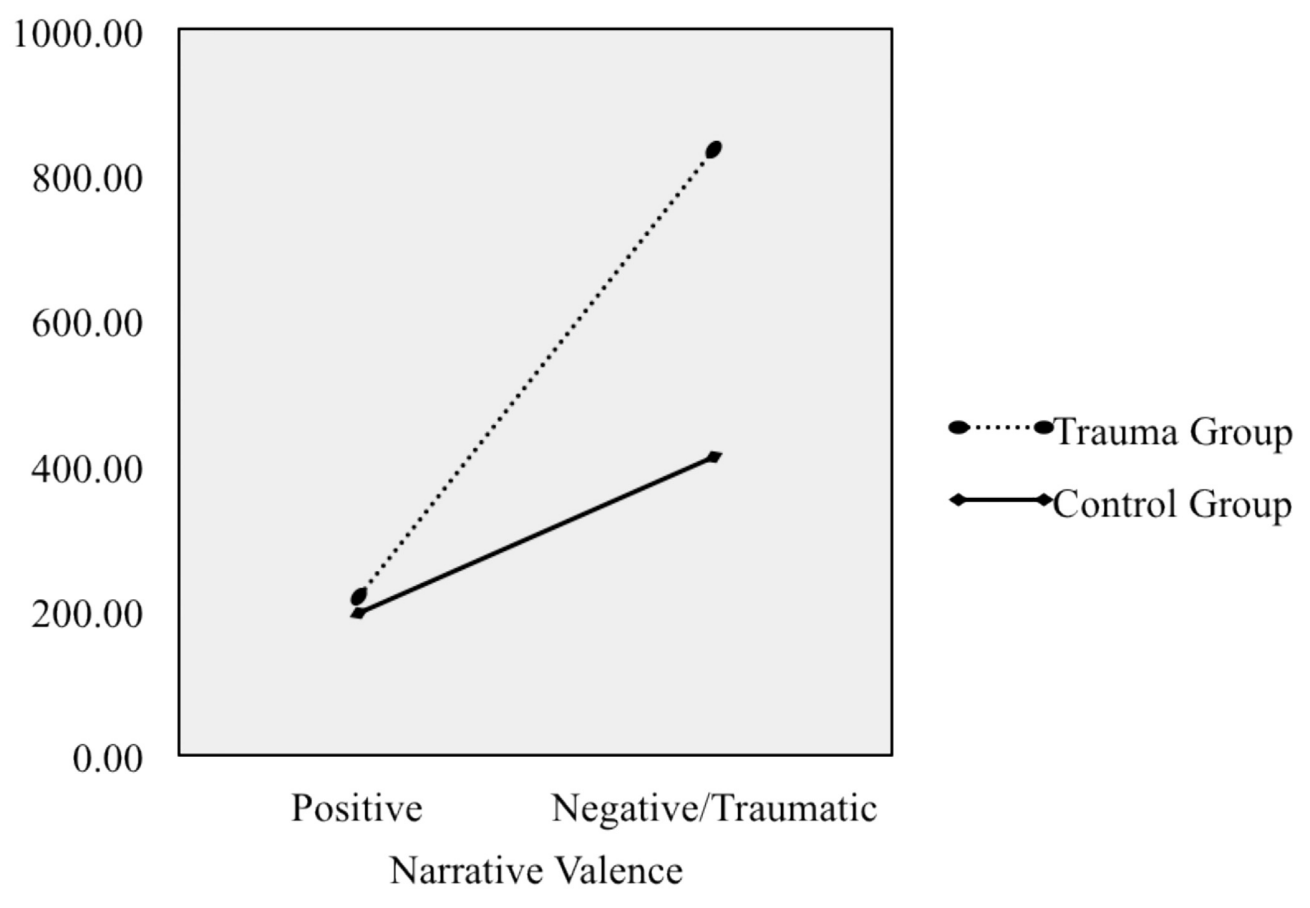
Marginal means for trauma and control groups in narrative length (WC) across positive and traumatic/negative narratives (*n* = 100)

According to previous literature, ANCOVA analysis, with the narrative length for the neutral event as covariate, showed that the word count differed by Group, *F*(1,97) = 12.644, *p* = .001, η^2^_partial_ = .115; and by Valence, *F*(1,97) = 23.704, *p* = .001, η^2^_partial_ = .196 (Table 3). The narratives from the trauma group were longer than for the controls, and the positive narratives were shorter than the negative/traumatic narratives for the two groups. Furthermore, there was a significant effect of Group × Valence interaction, *F*(1,97) = 12.177, *p* = .001, η^2^_partial_ = .112, with high observed power (1 - *β* = .933). The *post hoc* tests with Bonferroni corrections revealed that the differences between the groups were only significant for the negative/traumatic narratives: the traumatic narratives by the trauma group were longer than the negative narratives by the controls (*p* < .001). Fig 1 shows the marginal means in the narrative length for the two groups across narratives. However, when the speech rate was analyzed, with the speech rate for the neutral event as a covariate, no significant differences were found, although the trauma group tended slightly to use more words per minute than the control group (Table 3).

Additionally, because there were differences between groups in marital status, this variable was also introduced as covariate in further analysis, showing that it did not affect the significance of the differences previously found.

### Relationship between traumatic narrative aspects and features of the event and memory

As shown in Table 4, the longer narratives for the traumatic event in the trauma group were significantly associated with less time since the event (*p* = .021) and less anxiety during disclosure (*p* = .034). For other measures, the correlations were not significant, although longer narratives were slightly related to an increase in memory elaboration (*p =* .066). The correlations showed also a significant and inverse association between speech rate and anxiety during disclosure (*p* = .004). Moreover, a lower number of words per minute was linked to more cognitive and behavioral avoidance (*p* = .006), but not avoidance thinking as assessed by the AMQ, and more peritraumatic dissociation (*p* = .012). Other correlations were not statistically significant.

**Table 4:** Correlations between narrative length (WC) and speech rate (W/min.) for traumatic narratives and traumatic event characteristics (*n* = 50).

Variables	WC Traumatic	W/min. Traumatic
Time since event	-.325	-.183
	*p* = .021	*p* = .204
Event centrality (CES)	.056	-.132
	*p* = .700	*p* = .362
Peritraumatic dissociation (PDEQ)	-.058	-.353
	*p* = .688	*p* = .012
Avoid thinking (AMQ)	-.178	.233
	*p* = .216	*p* = .104
Avoidance (EGEP)	-.198	-.384
	*p* = .173	*p* = .006
Memory elaboration (AMQ)	.264	.154
	*p* = .066	*p* = .291
Have talked about the event	.017	.025
	*p* = .909	*p* = .867
Frequency of talking	.145	.202
	*p* = .340	*p* = .183
Received psychological therapy	.044	-.053
	*p* = .760	*p* = .714
Months in therapy	-.265	-.180
	*p* = .082	*p* = .241
Anxiety during disclosure	-.303	-.407
	*p* = .034	*p* = .004

### Relationship between traumatic and positive narrative aspects and psychological symptoms

The correlations between narrative length and speech rate for positive narratives and emotional symptoms showed that both variables in the trauma group were inversely associated with PTSD, depression, and anxiety. Subsequently, more symptoms were related to a lower word count and fewer words per minute in the description of the positive event. Specifically, the correlations for narrative length and speech rate were significant for PTSD diagnosis (*r* = -.309, *p* = .029 for narrative length, and *r* = -.306, *p* = .030 for speech rate) and overall PTSD scores (*r* = -.380, *p* = .007, and *r* = -.401, *p* = .004), avoidance and numbing symptoms (*r* = -.381, *p* = .007, and *r* = -.328, *p* = .022, arousal symptoms (*r* = -.371, *p* = .009, and *r* = -.397, *p* = .005), anxiety (*r* = -.483, *p* < .001, and *r* = -.426, *p* = .002), and depression (*r* = -.402, *p* = .004, and *r* = -.454, *p* = .001). Additionally, a lower speech rate was associated with more re-experiencing symptoms (*r* = -.309, *p* = .029).

A lower word count for the traumatic event was significantly associated only with more PTSD diagnosis (*r* = -.333, *p* = .018). However, a lower speech rate was related to more overall PTSD scores (*r* = -.443, *p* = .001), re-experiencing (*r* = -.394, *p* = .005), avoidance and numbing, (*r* = -.445, *p* = .001), functioning (*r* = -.375, *p* = .007), anxiety (*r* = -.373, *p* = .008), and depression (*r* = -.402, *p* = .004).

Against expectations, the regression analyses showed that the traumatic narrative length did not allow for predicting psychological adjustment. Both overall PTSD symptoms and anxiety were predicted by the positive narrative length and speech rate for the traumatic narrative. For PTSD symptoms, the model explained 24.3% of the variance, for the most part due to the traumatic speech rate, *F*(2,46) = 8.724, *p* = .001; for anxiety, it described 27.3% of the variance, due primarily to the positive word count *F*(2,47) = 10.218, *p* < .001. The only predictor of depression was the positive narrative length, *F*(1,47) = 12.195, *p* = .001 (Table 5).

**Table 5:** Stepwise multiple linear regression analysis for psychological symptoms, narrative length and speech rate (*n* = 50).

	*β*	*t*	*p*
**Narrative Aspects as Predictors**			
*Overall PTSD Symptoms*			
W/min. Traumatic	-.373	-2.882	.006
WC Positive	-.290	-2.241	.030
Adjusted R^2^ = .243			
*Anxiety*			
WC Positive	-.417	-3.327	.002
W/min. Traumatic	-.272	-2.168	.035
Adjusted R^2^ = .273			
*Depression*			
W/min. Positive	-.454	-3.492	.001
Adjusted R^2^ = .189			
**Emotional Variables as Predictors**			
*WC Positive*			
Anxiety	-.494	-3.856	< .001
Adjusted R^2^ = .228			
*W/min*. *Positive*			
Depression	-.456	-3.471	.001
Adjusted R^2^ = .190			
*W/min*. *Traumatic*			
Overall PTSD symptoms	-.481	-3.723	.001
Adjusted R^2^ = .215			

The bidirectional effects were verified. For the positive narratives, narrative length was predicted by anxiety, *F*(1,46) = 14.872, *p* < .001, with 22.8% of the explained variance (for speech rate by depression, *F*(1,46) = 12.049, *p* = .001). The speech rate for traumatic events was predicted by the overall PTSD symptoms, *F*(1,46) = 13.859, *p* = .001. No predictors were found for the traumatic narrative length. However, due to its significant correlation with time since the event and anxiety during disclosure, both variables were introduced in the regression model, explaining the second to be a modest 9% of variance, *F*(1,45) = 5.575, *p* = .023. Additionally, because of its relationship with the speech rate of traumatic narratives, anxiety during disclosure, avoidance and peritraumatic dissociation were introduced as potential predictors with no significant results.

## Discussion

The present study first specifically explored the trauma narrative length in victims seeking treatment. We only have knowledge of a study by Beaudreau [3, 4] that has addressed this issue with a sample consisting of community-dwelling adults (only 5% of the sample fulfilled the criteria for PTSD). Nevertheless, Beaudreau [4] admitted that treatment-seeking individuals, because of their degree of distress and the impact of the event, represent a better sample to perform the narrative analysis. Moreover, this study incorporated the data of speech rate, and a control group, as well as control narratives of other emotional events. Consequently, the present study followed the recommendations by Römisch et al. [8] concerning the need to use clinical samples and control groups, and control narratives, in trauma memory research. Additionally, narrative skills, such as narrative style and verbal intelligence, were specifically controlled in this study.

Consistent with the results found in the study by Beaudreau [4], the trauma narratives were longer; the negative, not traumatic, memories were also longer compared with the narratives of positive and neutral events. Additionally, the trauma narratives by traumatized women had a mean number of words more than twice that the mean number of words observed in the negative narratives by controls. This finding supports the hypothesis that the high arousal experience might lead to detailed and vivid recollections of traumatic events [29]. However, the narrative length is not an indicator of memory accuracy, so the results do not prove that the traumatic memories are enhanced.

Focusing only on the traumatized participants, a relationship was found between the word count of traumatic narratives and the time since the event. Therefore, although trauma leaves a strong imprint on the individual’s autobiographical memory, there was a tendency to decrease the narrative length over time according to previous research (e.g., Dekel & Bonanno [13]), which supports the hypothesis of the decay of memories. Moreover, the speech rate of trauma narratives tended to be slower with time, although the results were not significant. Contrary to expectations, event centrality was not associated with the narrative aspects of traumatic recollections. A likely explanation is that all of the victims had requested some assistance for domestic violence, so that although there were differences in centrality among them, trauma played an important role in the life and identity of all of the participants. Additionally, no associations were found for memory elaboration, talking about the event, receiving psychological attention or months of therapy. These results did not seem to support the hypothesis proposed by Foa et al. [19]. However, consistent with these authors’ arguments, the participants with more anxiety during disclosure constructed shorter trauma narratives and tended to narrate the event in a slower manner. Gray and Lombardo [9] explained that psychological interventions could increase the comfort level during disclosure and decrease the anxiety level, resulting in a more complete narrative regarding the event. We suggest that imaginal reliving treatment used by Foa et al. [19] would impact on the narrative length by decreasing anxiety, whereas other types of psychological interventions would have different goals. Furthermore, in this sample, the majority (90%) of the women were receiving treatment at the present; hence, no conclusions can be drawn. Unexpectedly, avoidance and peritraumatic dissociation were not related to the traumatic narrative length; however, both variables were related to a decelerated speech rate. It is presumably because they reflect difficulties in encoding and accessing memories, as well as in speech planning, which would result in long pauses, in agreement with the observations of Hashemi et al. [21] and Römisch et al. [8].

When adaptation trajectories in battered women were analyzed, the results showed that shorter traumatic narratives were associated with more PTSD diagnostics. The results are in the opposite direction than those reported in the work by Römisch et al. [8] who found an association between the narrative length and the worst emotional state. However, it should be noted that these authors included in their analyses traumatized and non-traumatized participants (who indicated that the most distressing event was not traumatic). In this study, only traumatized women were considered. In fact, when analyzing the entire sample, the narrative length of both the negative and traumatic events was positively correlated to the posttraumatic symptoms.

Contrary to expectations, the narrative length for the happiest event was a better predictor for the individual’s mental health than traumatic narrative length. Longer positive narratives predicted lower levels of PTSD symptoms and anxiety. Additionally, the speech rate for traumatic narratives impacted psychological distress, whereas depression was predicted by the speech rate of the positive narrative. The analyses also showed that the relationship between the narrative aspects and the emotional state after trauma was reciprocal and bidirectional.

Altogether, the findings revealed that, although traditional research has focused on the traumatic narrative length, the speech rate (even though it tended to be correlated with it) could have a specific effect on the results. In accordance with the results of Römisch et al. [8], a decelerated speech might indicate the poor elaboration of traumatic memories that cognitive models attribute to PTSD. This finding was also supported by the inverse association observed between the speech rate and the peritraumatic dissociation. Additionally, the participants with slow speech showed more anxiety during disclosure and likely activated avoidance mechanisms to cope with their distress. This research also changed the focus toward the consideration of positive memories, more than distressing memories, in traumatized patients. The studies on depressed patients have consistently showed that they present memory biases with difficulties to access positive events of their past and to generate specific memories [34, 35]. Similarly, the experience of a traumatic event could break into the autobiographical memory system, leading to deficits in recalling positive emotional experiences. The victims with a worse adjustment after trauma could have this memory bias over time, whereas recovery would require elaborating traumatic memories but also memories of happy events. This finding has important implications for developing narrative clinical interventions for PTSD.

Nevertheless, the present study has several limitations. First, the sample was selective. The trauma group was homogeneous with regard to gender and type of event suffered. The focus on a specific class of traumatic experiences, however, is typical for other studies and helps prevent premature conclusions across types of events. Interpersonal violence is one of the most impactful traumatic events because of its chronicity and a feeling of betrayal of trust in others [36]. Furthermore, the study may have oversampled traumatized women with good ability to narrate their experience. The majority of the women was in or was ready to enter psychotherapy, and they voluntarily chose to participate in the present study. Thus, we cannot exclude the likelihood that the narratives were influenced by psychotherapies. Finally, it would have been desirable to collect follow-up data to examine the narrative changes over time.

## Conclusions

Data of this study showed that as a whole narratives of distressful events were longer (i.e. included more words) that those of positive relevant event; more specifically, narratives of violence episodes by intimate partner in battered women were longer than narratives of highly distressful life events in non-traumatized women. Even more, these differences did not related with difference in the speech rate (i.e. speed); actually speed showed high consistency both between types of narratives (positive vs. negative/traumatic) and groups (trauma vs. no-trauma).

Among battered women, longer narratives of trauma were related to less anxiety during disclosure, and less time since the episode narrated, but not with other features such as centrality of the event, avoidance, or frequency of talking about the event; moreover, a quicker narration of the episode of violence was associated to less anxiety during disclosure, higher peritraumatic dissociation and more avoidance responses. Consequently, these aspects should to be taken into account when analyzing trauma narratives in this type of victims.

Furthermore, the study revealed some relationships among narrative length and speech rate, both for trauma and positive events, and posttraumatic, anxiety and depression symptoms in women victims of repeated violence by their intimate partners. Interestedly, narrative length and speech rate for positive events showed higher association with symptoms severity that those for trauma event; even more, speech rate was more relevant than narrative length when trauma narrative was considered.

All in all this study underlined the need to advance the study of narrative length but also of speech rate in both traumatic and positive narratives. Future research should explore linguistic aspects of narrative construction across different populations and autobiographical memories with different emotional impact. The way in which the victims express their personal experiences offers valuable information to gain a better understanding of the memory processes involved in the different adaptation trajectories after trauma.
